# Time Spent on Social Media and Associations with Mental Health in Young Adults: Examining TikTok, Twitter, Instagram, Facebook, Youtube, Snapchat, and Reddit

**DOI:** 10.1007/s41347-024-00474-y

**Published:** 2025-01-13

**Authors:** Matthew J. Woodward, Caitlin R. McGettrick, Olivia G. Dick, Mawsoof Ali, Jenni B. Teeters

**Affiliations:** Department of Psychological Sciences, Western Kentucky University, 1906 College Heights Boulevard, Bowling Green, Kentucky, 42101 USA

**Keywords:** Social media, TikTok, YouTube, Snapchat, Mental health, Anxiety

## Abstract

Time spent on social media has been an inconsistent predictor of mental health outcomes in young people. However, most studies have assessed social media use globally, with few investigations of the relative influence of specific social media platforms, which may partially account for mixed findings. Furthermore, studies often focus on a single mental health outcome, limiting understanding of how social media relates to psychological well-being. The purpose of the current study was to examine associations between time spent on multiple popular social media platforms and a variety of mental health-related outcomes in a sample of young adults. Participants included 575 young adults who completed an online survey assessing self-reported time spent on Twitter, TikTok, YouTube, Instagram, Reddit, Snapchat, and Facebook as well as depression, anxiety, PTSD, loneliness, friend support, and self-esteem. Path analyses showed that in the overall sample, greater use of Tiktok and YouTube were consistently associated with more mental health issues, whereas greater use of Snapchat was associated with fewer mental health issues. Models examining results for men and women separately suggested that use of Tiktok was more relevant in women’s mental health, whereas use of Reddit was more relevant in men’s mental health. Findings highlight that associations are not uniform across social media platforms. More research is needed that compares individual platforms and their relationship to psychological well-being as well as future studies examining how gender impacts findings.

## Time Spent on TikTok, Twitter, Instagram, Facebook, YouTube, Snapchat, and Reddit and Associations with Mental Health in Young Adults

Social media usage has increased rapidly in recent years. From 2005 to 2015, the percentage of U.S. young adults (i.e., ages 18 to 29) who endorsed using social media grew from 12 to 90% (Perrin, 2015). Rates of social media use are even more dramatic in younger cohorts, as 93% of teens and young adults report some level of social media use, and 81% report daily use (Rideout & Fox, 2018). Specifically, YouTube, Snapchat, Twitter, Instagram, TikTok, and Facebook are some of the most widely used social media sites among people below the age of 30 (Auxier & Anderson, 2021; Vogels et al., 2022). Concurrently, adolescents and young adults have reported increased mental health difficulties over the past decade compared to previous generations. From 2012 to 2018, depressive symptoms steadily increased in U.S. adolescents (Keyes et al., 2019), and from 2005 to 2014 the 12-month prevalence of major depressive episodes increased by 2.6% in adolescents and 0.8% in young adults (Mojtabai et al., 2016).

Because of the alarming increase in reported mental health difficulties among adolescents and young adults, there has been considerable interest in understanding the effects that social media has on young people’s psychological well-being. In particular, time spent on social media is the main facet of social media use that has received the most attention in empirical research. Overall, results in the literature have been mixed regarding the association between time spent using social media and mental health outcomes. Multiple studies have found a significant, albeit typically small, association between greater use of social media and worse mental health symptoms (Keles et al., 2020). A daily-diary study by Price and colleagues (2022) in young adults found that more social media use was associated with increased depression and PTSD over a one-month period. Furthermore, a randomized controlled trial by Lambert and colleagues (2022) found that individuals assigned to stop using social media for one week exhibited greater well-being and lower anxiety and depression compared to individuals randomized to continue using social media as usual. Additionally, one meta-analysis found a small but positive association among twelve studies examining adolescent social media use and depression (Ivie et al., 2020). Several other studies have also found links between social media use and mental health-related problems (e.g., Brunborg et al., 2022; Nesi et al., 2021).

On the other hand, several studies have failed to identify a significant relationship between social media use and worse mental health. Coyne and colleagues (2020) conducted an eight-year longitudinal study with 500 adolescents and young adults and did not find that increased use of social media was associated with depression or anxiety. Another study found that within-person increases in the amount of time spent on social media were generally not associated with worsened psychological distress, including depression, anxiety, and social isolation (Sewall et al., 2022). Additional studies have found similar results (Tang et al., 2021).

One factor that may account for this mixed pattern of results is that most studies to date in this literature have either focused on social media use broadly or examined only a singular social media platform (e.g., Ivie et al., 2020; Lup et al., 2015; Mackson et al., 2019; Tang et al., 2021). Notably, there are few studies comparing the relative influence of specific social media platforms on mental health-related problems. Aggregating time spent on social media is limiting as individuals often use a variety of different social media platforms, which fails to account for the heterogenous effects that different social media platforms might have. This limitation hampers understanding of the unique effects of specific social media platforms and could partly explain why global time spent on social media has been an inconsistent predictor of mental health difficulties. Additionally, studies that have focused on a single platform have primarily focused on older platforms such as Facebook and Instagram, with few examinations of more recent platforms such as TikTok, one the fastest growing social media apps among young people (Song et al., 2014; Vogels et al., 2022).

It is reasonable to believe that social media platforms may differ in their relationship with mental health outcomes as a plethora of social media apps exist that vary significantly in their design, function, and associated social networks. Although there is a dearth of empirical studies comparing the impact of different social media apps, a 2017 report in the United Kingdom surveyed 1,479 adolescents and young adults about their perceptions of how popular social media platforms including YouTube, Twitter, Facebook, Snapchat, and Instagram affected their well-being (Royal Society for Public Health, 2017). On average, participants reported that YouTube had a positive effect on their mental health, whereas the other platforms were rated as having a neutral or negative effect. Additionally, Perlis and colleagues (2021) examined whether use of Facebook, Instagram, LinkedIn, Pinterest, TikTok, Twitter, Snapchat, and YouTube predicted subsequent increases in depression in a sample of adults, finding that Snapchat, Facebook, and TikTok were associated with increased depression. However, this study only examined absence or presence of use rather than amount of time spent on the platforms and only focused on depression. Nevertheless, this suggests social media apps may be differentially related to psychological well-being, and more studies are needed that compare the influence of specific social media platforms and their relationship with mental health-related outcomes.

It is also relevant to examine a range of psychological outcomes when studying social media use, as social media use has been linked to an array of psychological difficulties and the influence of specific social media platforms may not be uniform across all mental health-related issues (e.g., Kelly et al., 2018; Perlis et al., 2021; Thai et al., 2023). Although internalizing problems such as anxiety, depression, and PTSD are some of the most common outcomes examined, social media use has also been linked with broader psychological difficulties such as low self-esteem (Kelly et al., 2018; O’Day & Heimberg, 2021). Moreover, because these platforms are social in nature, studies have also investigated the relationship between social media use and interpersonally-oriented outcomes. For example, social media use has been linked with increased feelings of social isolation (Lisitsa et al., 2020). However, social media use may not be exclusively harmful, as research indicates that adolescents and young adults frequently use social media as a way to build and maintain friendships and greater use has been linked with higher social support (Anderson, & Jiang, 2018; Kim, 2014). Furthermore, studies have suggested that the relationship between social media use and mental health may vary by gender (Tang et al., 2021; Twenge & Farley, 2021). Although findings are mixed in this area, some studies have found stronger effects for women (Heffer et al., 2019; Svensson et al., 2022), while others have found stronger effects for men (Houghton et al., 2018). Accordingly, it may be relevant to examine findings across men and women.

The primary aim of the current study was to examine the association between time spent on multiple popular social media platforms and mental health-related outcomes in a sample of young adults. Young adults represent an ideal sample to conduct this line of research given that they are prominent users of social media and are particularly at risk for experiencing mental health-related difficulties relative to other age groups (Rideout & Fox, 2018; SAMHSA, 2022). A second aim of the study was to examine findings separately among men and women to examine whether the influence of specific social media platforms on mental health varied across genders. It was hypothesized that use of Snapchat, Facebook, and TikTok would be associated with worse mental health, based upon findings from a prior study that examined linkages between several different social media platforms and depression (Perlis et al., 2021). It was also hypothesized that effects would be more robust for women relative to men given findings from previous work indicating that social media use and internalizing symptoms were more closely associated among girls than boys (Svensson et al., 2022; Twenge & Farley, 2021).

## Method

### Participants

Participants were 575 young adults at a large U.S. public university who received course credit for completing an online survey. Participants read the informed consent document and agreed to participate in the study, and the survey was administered through Qualtrics. Inclusion criteria were being ages 18 to 25 and ability to understand written English.

The sample initially included 601 individuals who completed the survey. Fifteen individuals were removed for failing multiple attention checks. An additional eleven individuals were excluded who were over the age of 25, resulting in a final sample of 575 participants. The mean age of participants was 19.1 (*SD* = 1.3). The sample was predominantly Caucasian (80.1%), followed by African American (11.7%), Hispanic or Latinx (5.2%), Asian American (4.2%), or another ethnicity (4.2%). The sample was mostly female (77.7%), followed by male (17.7%), non-binary (1.6%), and another unlisted gender (0.7%). Of the 575 participants, 78.8% identified as straight, 10.6% identified as bisexual, 3.1% identified as gay or lesbian, 2.3% identified as pansexual, 1.2% identified as queer, and 1.8% identified as asexual or another unlisted sexuality. No differences were found between men and women on age, ethnicity, or sexual orientation (see Table 1 for additional sample characteristics).

**Table 1:** Sample Descriptors

	Overall Sample	Women	Men		
	(*N* = 575)	(*n* = 447)	(*n* = 102)	Statistic	
Age (years)	19.05 (1.30)	19.00 (1.27)	19.27 (1.46)	*t* = −1.88, *p* = 0.06	
Race/Ethnicity^1^				χ^2^ = 1.70, *p* = 0.19	
Caucasian	80.1%	82.8%	76.5%		
African American	11.7%	10.1%	20.6%		
Hispanic or Latinx	5.2%	5.4%	5.9%		
Asian American	4.2%	5.1%	1.0%		
Other Ethnicity	4.2%	4.2%	4.0%		
Gender					
Female	77.7%				
Male	17.7%				
Non-Binary	1.6%				
Another Gender	0.7%				
Sexual Orientation				χ^2^ = 0.76, *p* = 0.39	
Straight	78.8%	81.7%	85.3%		
Gay or Lesbian	3.1%	1.6%	9.8%		
Bisexual	10.6%	11.9%	2.9%		
Pansexual	2.3%	1.8%	2.0%		
Queer	1.2%	1.1%	0.0%		
Asexual/Other	1.8%	2.0%	0.0%		
Social Media (hours per day)					
Facebook	0.62 (0.97)	0.70 (1.03)	0.28 (0.52)	*t* = 4.05, *p* < .001	
Instagram	1.55 (1.30)	1.58 (1.29)	1.35 (1.28)	*t* = 1.63, *p* = .10	
Reddit	0.04 (0.22)	0.02 (0.17)	0.13 (0.34)	*t* = −4.50, *p* = .003	
Twitter	0.43 (0.88)	0.41 (0.85)	0.44 (0.77)	*t* = -.28, *p* = .76	
TikTok	2.19 (1.87)	2.36 (1.83)	1.25 (1.61)	*t* = 5.61, *p* < .001	
Snapchat	2.43 (1.96)	2.45 (1.95)	2.27 (1.88)	*t* = 0.86, *p* = .38	
YouTube	1.63 (1.85)	1.45 (1.77)	2.27 (1.95)	*t* = −4.15, *p* < .001	
Total Hours/Day	8.86 (5.07)	8.94 (5.10)	7.95 (4.60)	*t* = 1.80, *p* = .07	

*Note*. Numbers in parentheses represent the standard deviation. ^1^Percentages sum to more than 100% due to individuals being able to select multiple categories

### Measures

#### Demographics

Participants completed a brief demographics measure assessing age, ethnicity, gender, and sexual orientation.

#### Social Media Use

Participants were asked to self-report on an average day the amount of time spent using multiple popular social media platforms, including Facebook, Instagram, Twitter, Reddit, TikTok, Snapchat, and YouTube. Response options were given in half-hour increments.

#### Potential Trauma Exposure

The Life Events Checklist for DSM-5 (LEC-5; Grey et. al., 2004; Weathers et. al., 2013a) was used to evaluate whether a participant had experienced a number of potentially traumatic events such as assault and natural disasters. Participants selected from a variety of 16 specified experiences whether the event ‘Happened to me’, ‘Witnessed it’, ‘Learned about it’, ‘Part of my job’, ‘Not sure’, or ‘Doesn't apply’.

#### PTSD

The PTSD Checklist (PCL-5; Weathers et al., 2013b) is a common 20-item self-report measure of PTSD symptoms. Symptoms are rated on a scale from 0 (“not at all”) to 4 (“extremely”) and a total score represents overall PTSD symptom severity, with greater scores representing higher PTSD symptoms. Participants were instructed to complete the PCL-5 related symptoms experienced in the past month. Scores range from 0 to 60. Cronbach’s alpha for the total score was high (α = 0.95). Only responses from individuals who endorsed at least one potentially traumatic event on the Life Events Checklist for DSM-5 (Weathers et. al., 2013a; *n* = 538) were included.

#### Depression

Depression symptoms were assessed using the Patient Health Questionnaire (PHQ-9; Kroenke et al., 2001). The PHQ-9 assesses frequency of depressive symptoms over the past two weeks with four response categories: 0 for ‘not at all’, 1 for ‘several days’, 2 for ‘more than half the days’, and 3 ‘Nearly every day’. The total score of the measure ranges from 0 to 27, with higher scores indicating higher levels of depression symptoms. The PHQ-9 has illustrated good internal consistency and test–retest reliability (Kroenke et al., 2001). Cronbach’s alpha was high for depression symptoms (α = 0.90).

#### Anxiety

Trait anxiety was assessed using the trait anxiety subscale of the State-Trait Anxiety Inventory (STAI; Spielberger et al., 1983). The STAI consists of 40 items and two subscales assessing both in-the-moment (i.e., state) and enduring (i.e., trait) anxiety. The measure has demonstrated good internal consistency and test–retest reliability studies (Spielberger et al., 1983;). Responses for the trait anxiety subscale assessed the frequency of feelings “in general”. Symptoms are rated on a scale of 1 (“almost never”) to 4 (“almost always”). Scores on the trait subtest range from 20 to 80 and higher scores are indicative of higher levels of trait anxiety, Cronbach’s alpha for the trait anxiety subscale was high (α = 0.93).

#### Loneliness

The UCLA Loneliness Scale (ULS-6; Neto, 2014) is a 6-item self-report measure of loneliness. Symptoms are rated on a scale from 1 (“I never feel this way”) to 4 (“I often feel this way”), with higher scores representing higher levels of loneliness. The measure has demonstrated a high level of internal consistency and criterion-related validity (Russell, 1996). Cronbach’s alpha for the total score was 0.83.

#### Self-Esteem

Self-Esteem was assessed using the Rosenberg Self-Esteem Inventory (RSE; Rosenberg, 1965): a common 10-item measure designed to assess self-esteem. This scale consists of five positively worded and five negatively worded items and higher scores reflect lower self-esteem. The RSE demonstrates excellent internal consistency and reliability (Schmitt & Allik, 2005; Torrey, 2000). Cronbach’s for the current study was excellent (α = 0.91).

#### Friend Support

Friend support was assessed using the Multidimensional Scale of Perceived Social Support (MSPSS; Zimet et al., 1988). This measure is a 12-item self-report with three subscales assessing perceptions of support from friends, family, and significant others. The items are scored on a 7-point Likert scale ranging from 1(“Very Strongly Disagree”) to 7 (“Very Strongly Agree”) with higher scores reflecting higher levels of perceived support. The friend subscale of the MSPSS demonstrates good internal consistency (0.85) and test–retest reliability (0.75). Cronbach’s alpha was high for friend support in the current study (α = 0.94).

## Procedure

All procedures conducted within this study were approved by the local Institutional Review Board. Data was collected from a university located in the midwestern United States. Prior to beginning the online survey, participants were provided with a consent form regarding confidentiality of responses and the ability to withdraw from the study at any point without penalty. Once participants agreed to participate, they completed the online survey and received course credit upon completion as compensation.

## Data Analytic Procedures

Data were initially screened for responses suggestive of random or invalid responding via review of random attention checks embedded throughout the survey. Before primary data analyses, data were examined for issues related to normality, including skew, kurtosis, and univariate and multivariate outliers, following recommendations from Tabachnick and Fidell (2019). No problems related to skew, kurtosis, or multivariate outliers were observed. A small number of univariate outliers were identified in the data and were winsorized. Due to a procedural error, the trait anxiety subscale of the STAI was administered only to a subset of the sample (n = 245). Multiple imputation was used to generate scores on individuals with missing responses for this subscale, and comparison of analyses using imputed data vs. non-imputed data showed virtually identical results.

Path analyses were in run in Mplus (v. 8.6). Path models included all exogenous (i.e., time on social media apps) and endogenous (i.e., mental health-related) variables in a single model. Paths were specified from each social media app to each mental health-related outcome. The various platforms were included in the same model to assess which platforms showed the most robust effects while accounting for the influence of other social media platforms. Covariances between all social media apps, as well as covariances between the residuals of all mental health-related outcomes, were specified. Acceptable model fit was examined using traditional model fit indices and was established by a non-significant chi-square value, an RMSEA value smaller than 0.08, a CFI and TLI value greater than 0.90, and an SRMR smaller than 0.10 (Bentler, 1990). Initial models were just-identified. In order to over-identify the model to examine model fit, one of the non-significant paths (i.e., Instagram → social support from friends) was randomly selected and fixed to 0.

Two path models were run. The first model was analyzed in the entire sample that included all genders and thus identified optimal path estimates irrespective of gender. After establishment of acceptable model fit, a second path model was run in which path estimates were allowed to vary between men and women. As such, this model identified optimal path estimates for men and women separately. Path models were run that included age, ethnicity, and sexual orientation as covariates; however, inclusion of these demographic variables resulted in a similar pattern of findings as models without covariates. For model parsimony, and in accordance with guidelines from Spector and Brannick (2011) regarding the misuse of demographic variables as covariates, the final models did not include these demographic control variables.

## Results

### Sample Characteristics

Examination of overall sample characteristics revealed that participants reported spending the most time on Snapchat (*M* = 2.4 h per day, *SD* = 2.0), followed by TikTok (*M* = 2.2 h per day, *SD* = 1.9), YouTube (*M* = 1.6 h per day, *SD* = 1.8), Instagram (*M* = 1.6 h per day, *SD* = 1.3), Facebook (*M* = 0.6 h per day, *SD* = 1.0), Twitter (*M* = 0.4 h per day, *SD* = 0.9), and Reddit (*M* = 0.04 h per day, *SD* = 0.2). The average total time spent on all social media platforms combined was over eight hours per day (*M* = 8.9 h, *SD* = 5.1). Independent samples t-tests comparing men and women on social media use showed that women spent significantly more hours per day than men on Facebook (*M* = 0.7, *SD* = 1.0 vs. *M* = 0.3, *SD* = 0.5; *p* < 0.001) and TikTok (*M* = 2.4 h per day, *SD* = 1.8 vs.* M* = 1.3, *SD* = 1.6; *p* < 0.001). Men spent more time per day than women on Reddit (*M* = 0.1, *SD* = 0.3; *M* = 0.02, *SD* = 0.2, *p* = 0.003) and YouTube (*M* = 2.3, *SD* = 2.0; *M* = 1.4, *SD* = 1.8, *p* < 0.001). See Table 1 for additional sample characteristics and gender comparisons.

### Results from the Overall Sample

**Fig. 1:**
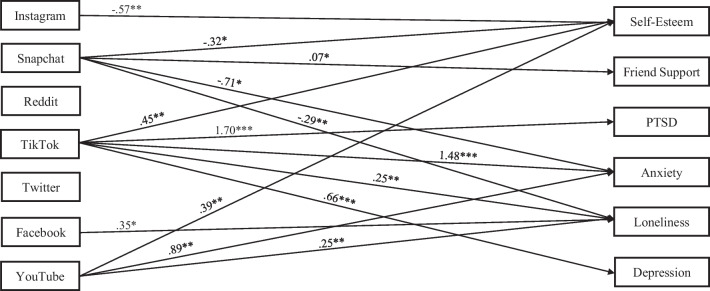
Unstandardized Coefficients from the Model Examining Associations Between Time Spent on Social Media Platforms and Mental Health-Related Outcomes in the Overall Sample. Note. For ease of visualization, non-significant paths, as well as covariances among exogenous variables and covariances among residuals of endogenous variables, are omitted from the figure

Results for the overall sample model are presented in Fig. 1, and path coefficients across samples are presented in Table 2. Unstandardized coefficients are presented for their ease of interpretation. Good model fit was found for the model in the combined sample of men and women, χ^2^(1) = 0.23, *p* = 0.63, RMSEA = 0.00 (90% CI = 0.00 – 0.09), CFI = 1.00, TLI = 1.00, and SRMR = 0.001. Regarding mental health symptoms, only use of Tiktok was associated with PTSD symptoms (B = 1.70, *p* = 0.003) and depression (B = 0.66, *p* = 0.01). A broader array of social media platforms were associated with trait anxiety, with use of Tiktok (B = 1.48, *p* < 0.001,) and YouTube (B = 0.89, *p* = 0.004) positively associated with trait anxiety, whereas use of Snapchat was negatively associated with trait anxiety (B = −0.29, *p* = 0.002). For interpersonal-related outcomes, use of Tiktok (B = 0.25, *p* = 0.01), YouTube (B = 0.25, *p* = 0.005), and Facebook (B = 0.35, *p* = 0.04) showed positive associations with loneliness, whereas use of Snapchat demonstrated negative associations with loneliness (B = −0.29, *p* = 0.002). Only use of Snapchat was linked with social support from friends, with more time on Snapchat associated with higher reported levels of perceived friend support (B = 0.07, *p* = 0.02). For self-esteem, greater use of TikTok (B = 0.45, *p* = 0.005) and YouTube (B = 0.39, *p* = 0.007) were positively associated with lower self-esteem (i.e., worse self-esteem), whereas greater use of Instagram (B = −0.57, *p* = 0.01) and Snapchat (B = −0.32, *p* = 0.03) were negatively associated with low self-esteem (i.e., more use was associated with higher self-esteem). No significant effects were observed for Twitter or Reddit.

**Table 2:** Unstandardized Coefficients from the Models for Men, Women, and the Entire Sample, Respectively

	**Outcomes**									
	***Self-Esteem***	***Friend Support***		***Loneliness***		***PTSD***			***Anxiety***	***Depression***
**Predictors**										
Facebook	-.16, .33, .46		.12, .05, .06		.36, .27, .35*		.13, .71, .41	-.87, 1.04, 1.23,		−1.46, .17, .08
Instagram	-.23, -.68**, -.57*		0^t^, 0^t^,0^t^		.12, -.31*, -.24		3.84*, −1.64*, -.52	-.21, -.96, -.72		.49, -.46, -.21
Reddit	2.42, 2.81, 2.06		.08, .13, .13		-.21, -.31, -.80		12.44*, .47, 2.32	6.31, 3.19, 2.80		4.02*, .55, 1.09
Twitter	.001, -.16, -.17		-.18, .11, .06		.25, -.09, -.15		2.37, -.09, -.49	.24, -.64, -.81		.95, .06, -.06
Tiktok	.06, .35, .45**		-.03, -.07*, -.05		-.18, .19, .25*		-.57, 1.71**, 1.70***	.23, 1.23**, 1.48***		-.23, .66***, .66***
Snapchat	-.19, -.14, -.32*		-.01, .09**, .07*		-.34, -.28*, -.29**		-.18, -.57, -.40	-.57, -.59, -.71*		-.30, -.12, -.17
YouTube	.66*, .38*, .39**		-.05, -.04, -.04		.49*, .31**, .25**		.82, .57, .41	1.34*, 1.08**, .90**		.42, .25, .19
***Total R***^***2***^	.08, .04*, .05**		.02, .04*,.02		.09, .05**,.05**		.12*, .03*, .03*	.12, .08**, .09***		.11, .04*, .04*

Note. Estimates are ordered such that the coefficients correspond to results from the men, women, and entire sample path models. *p < .05; **p < .01; ***p < .001. ^t^These paths were fixed to zero to overidentify the path models

### Results from the Model Examining Men and Women Separately

Good model fit was also found for the model in which paths were estimated separately for men and women, χ^2^(2) = 0.62, *p* = 0.74, RMSEA = 0.00 (90% CI = 0.00 – 0.08), CFI = 1.00, TLI = 1.00, and SRMR = 0.001. For women, only use of Tiktok was significantly associated with PTSD symptoms (B = 1.71, *p* = 0.001) and depression (B = 0.66, *p* < 0.001). Use of Tiktok (B = 1.23, *p* = 0.001) and YouTube (B = 1.08, *p* = 0.003) were positively associated with trait anxiety. Use of YouTube (B = 0.31, *p* = 0.004) showed positive associations with loneliness, whereas use of Snapchat (B = −0.28, *p* = 0.01) and Instagram (B = −0.31, *p* = 0.03) demonstrated negative associations with loneliness. Use of Snapchat (B = 0.09, *p* = 0.003) was positively associated with social support from friends, whereas use of Tiktok (B = −0.07, *p* < 0.05) was negatively associated with support from friends. For self-esteem, greater use of YouTube (B = 0.38, *p* = 0.02) was associated with lower self-esteem, whereas greater use of Instagram (B = −0.68, *p* = 0.004) was associated with higher self-esteem.

For men, greater use of Instagram (B = 3.84, *p* = 0.01) and Reddit (B = 12.44, *p* = 0.01) were associated with PTSD symptoms, whereas only use of Reddit (B = 4.02, *p* = 0.01) was associated with symptoms of depression. Only greater use of YouTube was linked with anxiety (B = 1.34, *p* = 0.03) and loneliness (B = 0.49, *p* = 0.01). No social media platforms were significantly associated with support from friends. For self-esteem, only greater use of YouTube (B = 0.65, *p* = 0.03) was associated with lower self-esteem.

## Discussion

The purpose of the current study was to examine associations between self-reported time spent on popular social media platforms and an array of mental health-related problems in a group of young adults. A second aim of the study was to examine findings separately among men and women to examine whether the influence of specific social media platforms on mental health varied across genders. Findings add to a growing body of literature documenting significant associations between increased use of social media use and worse mental health problems (Ivie et al., 2020; Song et al., 2014). However, results provide important new information as findings varied across both the social media platform and mental health outcome examined. Of note, several past studies have examined only a single mental health outcome (Perlis et al., 2021; Tang et al., 2021). Results from the current study highlight the importance of examining a range of mental health problems, as comorbidity of mental health conditions is common, and findings show the influence of social media is not uniform across all mental health problems (Kessler et al., 2011). However, the contribution of time spent on social media in predicting mental health problems in the sample was generally small, as altogether social media use typically explained less than 10% of the variance in each mental health problem across the sample (see Table 2). This is consistent with previous literature in this area demonstrating small effects (Ivie et al., 2020; Tang et al., 2021). As such, although social media may be one relevant contributor to mental health problems in young people, there are a multitude of important factors beyond social media that are important to examine in understanding mental health issues in young adults.

It is important to highlight that more time spent on social media was not universally associated with worse mental health. While greater use of Tiktok and YouTube were consistently associated with greater mental health problems, greater use of Snapchat was associated with fewer mental health-related difficulties (i.e., less anxiety and loneliness; more friend support and self-esteem). These findings are partially at odds with Perlis and colleagues (2021) study, which found that Snapchat, Facebook, and TikTok were associated with increases in depression, with no effects found for Instagram, Twitter, and YouTube. However, there were some notable differences between the Perlis et al. (2021) study and the current study. Perlis and colleagues used a significantly older sample with a mean age of 55, as opposed to the current study of young adults. Additionally, social media use was assessed as presence or absence of use rather than amount of time spent using each platform, and the study only focused on a sample of initially non-depressed adults. These methodological and demographic differences may account for the discrepancies in findings.

 Although the current study identified associations between several popular social media platforms and mental health-related difficulties, it is currently unclear the mechanisms that account for these associations, and there are a number of possible factors. A recent report by the Center for Countering Digital Hate (2022) showed that harmful content on Tiktok related to eating disorders and suicide is pushed to accounts within minutes of joining the app. Differences across platforms in how algorithms operate may influence the frequency and type of information with which users are exposed to that could have toxic effects on mental health. Additionally, research shows that one moderating factor in the relationship between social media use and mental health is the way that individuals engage with the platforms. Passive use (e.g., ‘doomscrolling’, lack of engagement in one-on-one exchanges with others online) has been linked with worse mental health, whereas active use (i.e., engaging directly with individuals online) has been linked with better mental health (Thorisdottir et al., 2019; Verduyn et al., 2021). It is possible that the nature and design of certain platforms may encourage problematic styles of use, which may account for discrepancies in their observed effects. However, studies have yet to investigate this area of inquiry. Conversely, the beneficial effects of social media on mental health in young people may stem from the ability to connect with others and in turn build social support, a factor which has been shown to consistently reduce risk of mental health issues (Harandi et al., 2017). This may account for the negative associations found between Snapchat use and mental health problems in the current study, as Snapchat was the only platform that was found to be associated with higher perceptions of friend support in the overall sample.

Taken altogether, the current results suggest that social media platforms vary in the positive and negative effects they have on young adults. This may partially explain the mixed findings observed in previous literature, particularly when studies aggregate social media use or assess use globally, which is common. The current study highlights the limitations of operationalizing social media use via a single indicator. Given significant differences in the design, nature, and social networks of each social media platform, it is important to examine the unique effects of individual platforms. More research is needed to compare the influence of individual platforms on mental health.

Findings support prior literature showing differences in associations between social media use and mental health across men and women (Tang et al., 2021; Twenge & Farley, 2021). Although some platforms were associated with mental health issues in both men and women (e.g., YouTube), other platforms were differentially associated with mental health across the two genders. Tiktok appeared to be more influential for women, whereas Reddit appeared to be uniquely associated with mental health problems in men. It is unclear if these differences are due to the platforms themselves affecting men and women differently or if they are a result of men and women gravitating to different platforms, as women in the sample spent more time on Tiktok, whereas men spent more time on Reddit. However, it should be noted that the platform that was used the most in the current study (i.e., Snapchat) was frequently linked with fewer mental health issues, indicating that it is likely not merely a function of spending greater time on a platform itself that is harmful. More research is needed on gender differences in social media usage and how this relates to mental health.

The current study adds to the body of literature on social media and mental health by being one of the first studies to examine use of an array of popular, contemporary social media platforms and their associations with a host of mental health difficulties in young adults. Results suggest that when working with young adults with mental health problems, there may be clinical utility not only in attending to the amount of time individuals spend on social media, but also which platforms individuals invest their time in. This recommendation is supported by previous research showing that individuals randomized to cease using Tiktok, Facebook, Twitter, and Instagram over one week experienced greater reductions in internalizing symptoms like anxiety and depression (Lambert et al., 2022). Whether these effects persist over the long term is unknown.

A number of limitations of the current investigation are relevant to consider. The current study was cross-sectional, and as such, is unable to elucidate the directionality of the observed associations. Although it is possible that use of social media causes or worsens existing mental health-related problems, it is also equally plausible that mental health problems encourage greater social media use. Although several associations were found between social media use and mental health problems, findings from the current study do necessarily indicate that use of these platforms played a causal role in mental health issues. Another limitation is that social media use was assessed via self-report. Although this is not uncommon, a recent meta-analysis found only moderate correlations between self-reported and objective measures of use (Parry et al., 2021). Additional investigations in this area would benefit from utilizing objective measures of social media use as well as comparing findings across objective and self-report measures of social media use. A final limitation is that the sample was largely Caucasian and female. Altogether, more comprehensive, longitudinal studies that assess a range of individual social media platforms across time using multiple modalities of assessment are warranted, especially among diverse samples. Given the results of the current study, additional studies would also benefit from examining possible gender differences when exploring dynamics between social media and mental health and elucidating the mechanisms accounting for these differences.

The rapid proliferation and complexities of unique and novel social media platforms presents a challenge in studying and understanding their linkages with mental health. Despite the contributions of the current investigation, findings do not resolve the debate regarding the impact of social media on mental health in young people. However, this study points to some fruitful ways for advancing research in this area, including a need for more examinations of individual social media platforms and inclusion of a multitude of mental health-related issues. Incorporating these elements into rigorous research methodology yields the greatest promise for generating accurate knowledge, educating the public, and guiding clinical intervention.

## Data Availability

The data supporting the findings in this study are available upon reasonable request from the corresponding author.

## References

[CR1] Anderson, M., & Jiang, J. (2018). *Teens, friendships and online groups*. Pew Research Center: Internet, Science & Tech. Retrieved October 10, 2023, from https://www.pewresearch.org/internet/2018/11/28/teens-friendships-and-online-groups/

[CR2] Auxier, B. & Anderson, M. (2021). *Social media use in 2021*. Pew Research Center. Retrieved September 17, 2023, from https://www.pewresearch.org/internet/2021/04/07/social-media-use-in-2021/

[CR3] Bentler, P. M. (1990). Comparative fit indexes in structural models. *Psychological Bulletin,**107*(2), 238–246. 10.1037/0033-2909.107.2.238 2320703 10.1037/0033-2909.107.2.238

[CR4] Brunborg, G. S., Skogen, J. C., & Burdzovic Andreas, J. (2022). Time spent on social media and alcohol use among adolescents: A longitudinal study. *Addictive Behaviors,**130*, 107294. 10.1016/j.addbeh.2022.107294 35231842 10.1016/j.addbeh.2022.107294

[CR5] Center for Countering Digital Hate. (2022). *Deadly by design*. Retrieved September 4, 2023, from https://counterhate.com/wp-content/uploads/2022/12/CCDH-Deadly-by-Design_120922.pdf

[CR6] Coyne, S. M., Rogers, A. A., Zurcher, J. D., Stockdale, L., & Booth, M. (2020). Does time spent using social media impact mental health? An eight-year longitudinal study. *Computers in Human Behavior,**104*, 106160. 10.1016/j.chb.2019.106160

[CR7] Gray, M. J., Litz, B. T., Hsu, J. L., & Lombardo, T. W. (2004). Psychometric properties of the life events checklist. *Assessment,**11*(4), 330–341. 10.1177/1073191104269954 15486169 10.1177/1073191104269954

[CR8] Harandi, T. F., Taghinasab, M. M., & Nayeri, T. D. (2017). The correlation of social support with mental health: A meta-analysis. *Electronic Physician,**9*(9), 5212–5222. 10.19082/5212 29038699 10.19082/5212 PMC5633215

[CR9] Heffer, T., Good, M., Daly, O., Macdonell, E., & Willoughby, T. (2019). The longitudinal association between social-media use and depressive symptoms among adolescents and young adults: An empirical reply to Twenge et al. (2018). *Clinical Psychological Science, 7*(3), 462–470. 10.1177/2167702618812727

[CR10] Houghton, S., Lawrence, D., Hunter, S. C., Rosenberg, M., Zadow, C., Wood, L., & Shilton, T. (2018). Reciprocal relationships between trajectories of depressive symptoms and screen media use during adolescence. *Journal of Youth and Adolescence,**47*(11), 2453–2467. 10.1007/s10964-018-0901-y 30046970 10.1007/s10964-018-0901-y PMC6208639

[CR11] Ivie, E. J., Pettitt, A., Moses, L. J., & Allen, N. B. (2020). A meta-analysis of the association between adolescent social media use and depressive symptoms. *Journal of Affective Disorders,**275*, 165–174. 10.1016/j.jad.2020.06.014 32734903 10.1016/j.jad.2020.06.014

[CR12] Keles, B., McCrae, N., & Grealish, A. (2020). A systematic review: The influence of social media on depression, anxiety and psychological distress in adolescents. *International Journal of Adolescence and Youth,**25*(1), 79–93. 10.1080/02673843.2019.1590851

[CR13] Kelly, Y., Zilanawala, A., Booker, C., & Sacker, A. (2018). Social media use and adolescent mental health: Findings from the UK millennium cohort study. *eclinicalmedicine,**6*, 59–68. 10.1016/j.eclinm.2018.12.005 31193561 10.1016/j.eclinm.2018.12.005 PMC6537508

[CR14] Kessler, R. C., Ormel, J., Petukhova, M., McLaughlin, K. A., Green, J. G., Russo, L. J., Stein, D. J., Zaslavsky, A. M., Aguilar-Gaxiola, S., Alonso, J., Andrade, L., Benjet, C., de Girolamo, G., de Graaf, R., Demyttenaere, K., Fayyad, J., Haro, J. M., Hu, C. yi, Karam, A., … Üstün, T. B. (2011). Development of lifetime comorbidity in the WHO World Mental Health (WMH) surveys. *Archives of General Psychiatry*, *68*(1), 90–10010.1001/archgenpsychiatry.2010.180 10.1001/archgenpsychiatry.2010.180 PMC3057480 21199968

[CR15] Keyes, K. K., Gary, D., O’Malley, P. M., Hamilton, A., & Schulenberg, A. (2019). Recent increases in depressive symptoms among US adolescents: Trends from 1991 to 2018. *Social Psychiatry and Psychiatric Epidemiology,**54*, 987–996. 10.1007/s00127-019-01697-8 30929042 10.1007/s00127-019-01697-8 PMC7015269

[CR16] Kim, H. (2014). Enacted social support on social media and subjective well-being. *International Journal of Communication,**8*, 2201–2221.

[CR17] Kroenke, K., Spitzer, R. L., & Williams, J. B. (2001). The PHQ-9: Validity of a brief depression severity measure. *Journal of General Internal Medicine,**16*(9), 606–613. 10.1046/j.1525-1497.2001.016009606.x 11556941 10.1046/j.1525-1497.2001.016009606.x PMC1495268

[CR18] Lambert, J., Barnstable, G., Minter, E., Cooper, J., & McEwan, D. (2022). Taking a one-week break from social media improves well-being, depression, and anxiety: A randomized controlled trial. *Cyberpsychology, Behavior, and Social Networking,**25*(5), 287–293. 10.1089/cyber.2021.0324 35512731 10.1089/cyber.2021.0324

[CR19] Lisitsa, E., Benjamin, K. S., Chun, S. K., Skalisky, J., Hammond, L. E., & Mezulis, A. H. (2020). Loneliness among young adults during COVID-19 pandemic: The mediational roles of social media use and social support seeking. *Journal of Social and Clinical Psychology,**39*(8), 708–726. 10.1521/jscp.2020.39.8.708

[CR20] Lup, K., Trub, L., & Rosenthal, L. (2015). Instagram #Instasad?: Exploring associations among instagram use, depressive symptoms, negative social comparison, and strangers followed. *Cyberpsychology, Behavior and Social Networking,**18*, 247–252. 10.1089/cyber.2014.0560 25965859 10.1089/cyber.2014.0560

[CR21] Mackson, S. B., Brochu, P. M., & Schneider, B. A. (2019). Instagram: Friend or foe? The application’s association with psychological well-being. *New Media & Society,**21*(10), 2160–2182. 10.1177/1461444819840021

[CR22] Mojtabai, R., Olfson, M., & Han, B. (2016). National trends in the prevalence and treatment of depression in adolescents and young adults. *Pediatrics*, *138*(6). 10.1542/peds.2016-1878 10.1542/peds.2016-1878 PMC5127071 27940701

[CR23] Nesi, J., Burke, T. A., Bettis, A. H., Kudinova, A. Y., Thompson, E. C., MacPherson, H. A., Fox, K. A., Lawrence, H. R., Thomas, S. A., Wolff, J. C., Altemus, M. K., Soriano, S., & Liu, R. T. (2021). Social media use and self-injurious thoughts and behaviors: A systematic review and meta-analysis. *Clinical Psychology Review*, 102038–102038. 10.1016/j.cpr.2021.102038 10.1016/j.cpr.2021.102038 PMC8243901 34034038

[CR24] Neto, F. (2014). Psychometric analysis of the short-form UCLA Loneliness Scale (ULS-6) in older adults. *European Journal of Ageing,**11*(4), 313–319. 10.1007/s10433-014-0312-1 28804337 10.1007/s10433-014-0312-1 PMC5549168

[CR25] O’Day, E. B., & Heimberg, R. G. (2021). Social media use, social anxiety, and loneliness: A systematic review. *Computers in Human Behavior Reports,**3*, 100070. 10.1016/j.chbr.2021.100070

[CR26] Parry, D. A., Davidson, B. I., Sewall, C. J. R., Fisher, J. T., Mieczkowski, H., & Quintana, D. S. (2021). A systematic review and meta-analysis of discrepancies between logged and self-reported digital media use. *Nature Human Behaviour,**5*(11), 1535–1547. 10.1038/s41562-021-01117-5 34002052 10.1038/s41562-021-01117-5

[CR27] Perlis, R. H., Green, J., Simonson, M., Ognyanova, K., Santillana, M., Lin, J., Quintana, A., Chwe, H., Druckman, J., Lazer, D., Baum, M. A., & Della Volpe, J. (2021). Association between social media use and self-reported symptoms of depression in US Adults. *JAMA Network Open,**4*(11), e2136113. 10.1001/jamanetworkopen.2021.36113 34812844 10.1001/jamanetworkopen.2021.36113 PMC8611479

[CR28] Perrin, A. (2015). *Social media usage: 2005–2015*. Pew Research Center. Retrieved August 27, 2023, from https://www.pewresearch.org/internet/2015/10/08/social-networking-usage-2005-2015/

[CR29] Price, M., Legrand, A. C., Brier, Z. M. F., van Stolk-Cooke, K., Peck, K., Dodds, P. S., Danforth, C. M., & Adams, Z. W. (2022). Doomscrolling during COVID-19: The negative association between daily social and traditional media consumption and mental health symptoms during the COVID-19 pandemic. *Psychological Trauma: Theory, Research, Practice, and Policy.,**14*(8), 1338–1346. 10.1037/tra0001202 35157484 10.1037/tra0001202 PMC10074257

[CR30] Rideout, V., & Fox, S. (2018). Digital health practices, social media use, and mental well-being among teens and young adults in the U.S. *Articles, Abstracts, and Reports,* 1093. Retrieved September 14, 2023, from https://digitalcommons.psjhealth.org/publications/1093

[CR31] Rosenberg, M. (1965). *Society and the adolescent self-image*. Princeton University Press.

[CR32] Royal Society for Public Health. (2017). *Social media and young people’s mental health and wellbeing*. Retrieved August 20, 2023, from https://www.rsph.org.uk/static/uploaded/d125b27c-0b62-41c5-a2c0155a8887cd01.pdf

[CR33] Russell, D. W. (1996). UCLA Loneliness Scale (Version 3): Reliability, validity, and factor structure. *Journal of Personality Assessment,**66*(1), 20–40. 10.1207/s15327752jpa6601_2 8576833 10.1207/s15327752jpa6601_2

[CR34] Schmitt, D. P., & Allik, J. (2005). Simultaneous administration of the Rosenberg Self-Esteem Scale in 53 nations: Exploring the universal and culture-specific features of global self-esteem. *Journal of Personality and Social Psychology,**89*(4), 623. 10.1037/0022-3514.89.4.623 16287423 10.1037/0022-3514.89.4.623

[CR35] Sewall, C. J. R., Goldstein, T. R., Wright, A. G. C., & Rosen, D. (2022). Does objectively measured social-media or smartphone use predict depression, anxiety, or social isolation among young adults? *Clinical Psychological Science,**10*(5), 997–1014. 10.1177/21677026221078309 36406004 10.1177/21677026221078309 PMC9671480

[CR36] Song, H., Zmyslinski-Seelig, A., Kim, J., Drent, A., Victor, A., Omori, K., & Allen, M. (2014). Does Facebook make you lonely? A meta-analysis. *Computers in Human Behavior,**36*, 446–452. 10.1016/j.chb.2014.04.011

[CR37] Spector, P. E., & Brannick, M. T. (2011). Methodological urban legends: The misuse of statistical control variables. *Organizational Research Methods,**14*(2), 287–305. 10.1177/1094428110369842

[CR38] Spielberger, C. D., Gorsuch, R. L., Lushene, R., Vagg, P. R., & Jacobs, G. A. (1983). *Manual for the State-Trait Anxiety Inventory (Form Y).* Consulting Psychologists Press, Inc.

[CR39] Substance Abuse and Mental Health Services Administration [SAMHSA]. (2022). *Key substance use and mental health indicators in the United States: Results from the 2021 National Survey on Drug Use and Health*. Retrieved September 10, 2023, from https://www.samhsa.gov/data/report/2021-nsduh-annual-national-report

[CR40] Svensson, R., Johnson, B., & Olsson, A. (2022). Does gender matter? The association between different digital media activities and adolescent well-being. *BMC Public Health,**22*(1), 273. 10.1186/s12889-022-12670-7 35144587 10.1186/s12889-022-12670-7 PMC8832746

[CR41] Tabachnick, B. G., & Fidell, L. S. (2019). *Using multivariate statistics. Pearson,**6*, 497–516. Retrieved July 21, 2023, from https://www.pearson.com/en-us/subject-catalog/p/using-multivariate-statistics/P200000003097/9780137526543

[CR42] Tang, S., Werner-Seidler, A., Torok, M., Mackinnon, A. J., & Christensen, H. (2021). The relationship between screen time and mental health in young people: A systematic review of longitudinal studies. *Clinical Psychology Review,**86*, 102021–102021. 10.1016/j.cpr.2021.102021 33798997 10.1016/j.cpr.2021.102021

[CR43] Thai, H., Davis, C. G., Mahboob, W., Perry, S., Adams, A., & Goldfield, G. S. (2023). Reducing social media use improves appearance and weight esteem in youth with emotional distress. *Psychology of Popular Media*10.1037/ppm0000460

[CR44] Thorisdottir, I. E., Sigurvinsdottir, R., Asgeirsdottir, B. B., Allegrante, J. P., & Sigfusdottir, I. D. (2019). Active and passive social media use and symptoms of anxiety and depressed mood among Icelandic adolescents. *CyberPsychology, Behavior & Social Networking,**22*(8), 535–542. 10.1089/cyber.2019.0079 31361508 10.1089/cyber.2019.0079

[CR45] Torrey, W. C., Mueser, K. T., McHugo, G. H., & Drake, R. E. (2000). Self-esteem as an outcome measure in studies of vocational rehabilitation for adults with severe mental illness. *Psychiatric Services,**51*(2), 229–233. 10.1176/appi.ps.51.2.229 10655008 10.1176/appi.ps.51.2.229

[CR46] Twenge, J. M., & Farley, E. (2021). Not all screen time is created equal: Associations with mental health vary by activity and gender. *Social Psychiatry and Psychiatric Epidemiology,**56*(2), 207–217. 10.1007/s00127-020-01906-9 32743778 10.1007/s00127-020-01906-9

[CR47] Verduyn, P., Gugushvili, N., & Kross, E. (2021). The impact of social network sites on mental health: Distinguishing active from passive use. *World Psychiatry,**20*(1), 133–134. 10.1002/wps.20820 33432746 10.1002/wps.20820 PMC7801842

[CR48] Vogels, E. A., Gelles-Watnick, R., & Massarat, N. (2022). *Teens, social media and technology 2022*. Pew Research Center. Retrieved August 12, 2023, from https://www.pewresearch.org/internet/

[CR50] Weathers, F. W., Blake, D. D., Schnurr, P. P., Kaloupek, D. G., Marx, B. P., & Keane, T. M. (2013a). *The life events checklist for DSM-5 (LEC-5)*. Retrieved August 1, 2020, from www.ptsd.va.gov

[CR51] Weathers, F. W., Litz, B. T., Keane, T. M., Palmieri, P. A., Marx, B. P., & Schnurr, P. P. (2013b). *The PTSD checklist for DSM-5 (PCL-5)*. Retrieved August 1, 2020, from www.ptsd.va.gov

[CR52] Zimet, G. D., Dahlem, N. W., Zimet, S. G., & Farley, G. K. (1988). The multidimensional scale of perceived social support. *Journal of Personality Assessment,**52*(1), 30–41. 10.1207/s15327752jpa5201_2 10.1080/00223891.1990.9674095 2280326

